# NURTURING INTERDISCIPLINARY EXCELLENCE: INSIGHTS FROM ADVANCED FELLOWSHIP IN GERIATRICS FELLOWS

**DOI:** 10.1093/geroni/igae098.0902

**Published:** 2024-12-31

**Authors:** Monica Serra, Sarah Wherry, Alexander Erickson, Yeonsu Song, Betsy Yang, Quratulain Syed

**Affiliations:** The University of Texas Health San Antonio, San Antonio, Texas, United States; VA Eastern Colorado Healthcare System, Aurora, Colorado, United States; VA Greater Los Angeles Healthcare System, Los Angeles, California, United States; University of California Los Angeles, Los Angeles, California, United States; Palo Alto VA Medical Center, Palo Alto, California, United States; Joseph Maxwell Cleland Atlanta VA Medical Center, Atlanta, Georgia, United States

## Abstract

This facilitated panel discussion offers a comprehensive exploration of the transformative impact of the Veterans Health Administration’s (VHA) Advanced Fellowship in Geriatrics across various disciplines within the post-graduate clinical and clinical research spectrum. Drawing from the perspectives of current and former fellows spanning diverse backgrounds including medicine, nursing, psychology, public health, and research, this session delves into their fellowship experiences, the array of activities they engaged in, and the profound influence of the fellowship on their professional trajectories. The Advanced Fellowship in Geriatrics provides a robust platform for fellows to engage in clinical rotations, honing their skills in geriatric assessment, management, and interdisciplinary collaboration. Additionally, through structured mentorship and access to research resources, fellows are empowered to conduct impactful clinical research, fostering their professional growth and contributing to the advancement of high-quality, patient-centered care within the VHA. Through a multi-disciplinary lens, panelists will describe their encounters experiences within the post-graduate training VHA fellowship, encompassing clinical rotations, research endeavors, educational initiatives, and quality improvement projects tailored to the unique needs of older adults and older veterans. By highlighting their collaborative endeavors and interdisciplinary interactions, the discussion will underscore the significance of a holistic approach in addressing the complex healthcare challenges faced by aging populations. Additionally, the panel will illuminate the instrumental role of the fellowship in enhancing healthcare delivery within the VHA, fostering innovation, and optimizing geriatric care practices to meet the evolving demands of veterans.

